# Fatality and risk features for prognosis in COVID-19 according to the care approach – a retrospective cohort study

**DOI:** 10.1371/journal.pone.0248869

**Published:** 2021-03-23

**Authors:** Mariano Andrés, Jose-Manuel Leon-Ramirez, Oscar Moreno-Perez, José Sánchez-Payá, Ignacio Gayá, Violeta Esteban, Isabel Ribes, Diego Torrus-Tendero, Pilar González-de-la-Aleja, Pere Llorens, Vicente Boix, Joan Gil, Esperanza Merino, on behalf of COVID19-ALC research group

**Affiliations:** 1 Department of Rheumatology, Alicante General University Hospital, Institute of Sanitary and Biomedical Research (ISABIAL), Alicante, Spain; 2 Department of Clinical Medicine, Miguel Hernández University, Elche, Spain; 3 Department of Pneumology, Alicante General University Hospital, Institute of Sanitary and Biomedical Research (ISABIAL), Alicante, Spain; 4 Department of Endocrinology and Nutrition, Alicante General University Hospital, Institute of Sanitary and Biomedical Research (ISABIAL), Alicante, Spain; 5 Department of Preventive Medicine, Alicante General University Hospital, Institute of Sanitary and Biomedical Research (ISABIAL), Alicante, Spain; 6 Department of Internal Medicine, Alicante General University Hospital, Institute of Sanitary and Biomedical Research (ISABIAL), Alicante, Spain; 7 Unit of Infectious Diseases, Alicante General University Hospital, Institute of Sanitary and Biomedical Research (ISABIAL), Alicante, Spain; 8 Parasitology Area, Miguel Hernández University, Elche, Spain; 9 Department of Emergency, Alicante General University Hospital, Institute of Sanitary and Biomedical Research (ISABIAL), Alicante, Spain; National Institute for Infectious Diseases Lazzaro Spallanzani-IRCCS, ITALY

## Abstract

**Introduction:**

This study analyzed the impact of a categorized approach, based on patients’ prognosis, on major outcomes and explanators in patients hospitalized for COVID-19 pneumonia in an academic center in Spain.

**Methods:**

Retrospective cohort study (March 3 to May 2, 2020). Patients were categorized according to the followed clinical management, as maximum care or limited therapeutic effort (LTE). Main outcomes were all-cause mortality and need for invasive mechanical ventilation (IMV). Baseline factors associated with outcomes were analyzed by multiple logistic regression, estimating odds ratios (OR; 95%CI).

**Results:**

Thirty-hundred and six patients were hospitalized, median age 65.0 years, 57.8% males, 53.3% Charlson index ≥3. The overall all-cause fatality rate was 15.0% (n = 46). Maximum care was provided in 238 (77.8%), IMV was used in 38 patients (16.0%), and 5.5% died. LTE was decided in 68 patients (22.2%), none received IMV and fatality was 48.5%. Independent risk factors of mortality under maximum care were lymphocytes <790/mm^3^, troponin T >15ng/L and hypotension. Advanced age, lymphocytes <790/mm^3^ and BNP >240pg/mL independently associated with IMV requirement.

**Conclusion:**

Overall fatality in the cohort was 15% but markedly varied regarding the decided approach (maximum care versus LTE), translating into nine-fold higher mortality and different risk factors.

## Introduction

Novel coronavirus disease 2019 (COVID-19), caused by SARS-CoV-2 virus, emerged in China, late in 2019 and has rapidly extended worldwide. Despite severity remains to be firmly set, grossly about 80% of infected people feel a mild or almost asymptomatic process, whereas 20% develop an inflammatory disease with major lung damage [1], and frequent involvement of other organs [2, 3]. Fatality may be high, but numbers dramatically vary from 1.4% to 28.3% [4, 5].

Ethnic and geographical variations are known to impact health outcomes and mortality [6]. Most case series coming from China [4, 5, 7–18], there is a paucity of data on European populations [19–21]. Independent risk factors of mortality, in Asians, are advanced age, cardiovascular disease, high SOFA score, high D-dimer and troponin I levels, and low CD3^+^CD8^+^ T lymphocyte counts [5, 11, 13]. Similar robust data from European populations are lacking. Furthermore, published data focus on COVID-19 inpatients outcomes as a whole, but how outcomes and risk factors can vary according to the approach based on patients’ characteristics, remains unknown.

This study aims to analyze the impact of a categorized approach on major outcomes and mortality risk factors in patients hospitalized for COVID-19 pneumonia in an academic Spanish center.

## Material and methods

### Patients and study design

This is a retrospective cohort study of patients with COVID-19 pneumonia hospitalized in an academic center of Spain. The study period was between March 3 and May 2, 2020, while the data analyses were finished at May 29, 2020. HGUA-ISABIAL ethics committee approved the study (exp. 200145); being retrospective, obtaining informed consent from participants was waived. The research was conducted according to the principles of the World Medical Association 2013 Declaration of Helsinki [22].

Potential candidates were identified either from discharge reports (provided by the Admission and Clinical Documentation unit) or from Microbiology and Preventive Medicine departments databases. COVID-19 diagnosis required being tested positive by reverse transcriptase–polymerase chain reaction (RT-PCR) for SARS-CoV-2, mainly in oropharyngeal aspirates; cases tested repeatedly negative for SARS-CoV-2, but high suspicion of COVID-19 by attending clinicians were also included. Criteria for hospital admission included advanced age, significant comorbidities, severe symptoms or poor clinical status, hypoxemia at room air (oximetry <94%, PaO2:FiO2 <300mmHg) and/or significant radiological pulmonary opacities (multilobar or bilateral opacities).

An agreed protocol of diagnosis and management was followed in clinical grounds to attend COVID-19 patients in the center. Patients were admitted to a multi-disciplinary especially created COVID-19 department. Involved specialties included respiratory medicine, infectious diseases, internal medicine, and volunteers from other fields. Daily meetings were undertaken to discuss complex cases and jointly decide therapeutic attitudes. Colleagues from intensive care also attended the meetings; management protocols were updated as needed.

Besides the general and respiratory support, pharmacological agents included hydroxychloroquine, alone or plus azithromycin and/or lopinavir-ritonavir, based on current knowledge and risk for QT prolongation. Intravenous tocilizumab (TCZ) was decided for severe cases at admission or in the case of rapid progression of respiratory failure, radiologic opacities or severe systemic inflammatory response during admission. Patients received an initial 600mg dose, with second or third doses (400mg) in the next 24h as needed; however, from March 30 on, the Spanish drug agency restricted TCZ to a single 600mg dose (400mg for bodyweight <75kg). If unsuccessful, the protocol established methylprednisolone 250mg/day for three days and, if required, subsequent individualized treatment with anakinra or immunoglobulins.

### Variables and data collection

#### Explanatory variables

Data on demographics, signs and symptoms, comorbidities and Charlson index, usual medications, imaging, laboratory and treatments were obtained from electronic medical records during admission and after discharge (all patients were followed by telephone for at least two weeks). Cases were considered nosocomial when symptoms developed <7days after a previous hospitalization or ≥7days after being admitted for other indication–this extended period aimed to cover the average incubation time of SARS-CoV-2 infection [23].

The study population was categorized into two groups of interest: *maximum care* (ICU and intubation as needed) and *limited therapeutic effort* -LTE- (no candidates to invasive ventilation [24–26]). The attending team agreed with the families the suitable approach for each individual, considering patients and disease characteristics (age, comorbidities, frailty, short pre-admission life expectancy, and extremely severe or advanced irreversible disease) and registered it in records. No predefined protocol for categorization was used. Antivirals, anti-inflammatories, and non-invasive ventilation were administered according to individual assessment.

#### Outcomes

a) All-cause mortality (either in-hospital or after discharge) and associated factors. Fatality rate was calculated as the number of deaths divided by the number of admitted cases of COVID-19 in the study period. COVID-19 related death was defined as progressive pneumonia leading to fatal respiratory failure, often complicated with vascular event (pulmonary embolism, myocardial infarction or stroke) [27]. b) The requirement of invasive mechanical ventilation (IMV) and associated factors.

### Statistical analysis

Categorical and continuous variables are given as frequencies (percentages) and as median (interquartile range), respectively. For logistic regression, continuous were categorized on their 75-percentiles within each population, to show the impact of severe, extreme values in the outcomes–except for those in which severity is defined by lowest levels, such as lymphocyte counts, where 25-percentiles were used. For the following variables, standard categorizations were followed: age ≥65years, Charlson comorbidity index ≥3, estimated glomerular filtration rate <60ml/min/1.73m^2^ (by CKD-EPI formula), oximetry <94% and PaO2:FiO2 <300mmHg [28], CURB65 score ≥3 [29], systolic and diastolic blood pressures <100 and 60mmHg, respectively, heart rate >100bpm and respiratory rate >24rpm.

Analyses were performed separately for the maximum care and LTE groups. Cumulative incidences of outcomes for each explanatory variable were registered. Associations were evaluated by chi-2 test. Multiple logistic regression models were built to explore risk factors at presentation associated with further mortality and use of IMV; odds ratios (OR) with 95% confidence intervals (95%CI) were estimated. Variables were included as covariates if shown significant associations in univariate models (P<0.050). Some covariates could be excluded in case of been highly correlated, >20% of missing values or number of events was too small to calculate odds ratios. Accordingly, in the maximum care, multivariate models for fatality and need of IMV included 184 and 186 participants, respectively. IBM SPSS Statistics v25 (Armonk, NY) was used for analyses. P<0.050 defined statistical significance. The dataset created and analyzed is available in an online repository [30].

## Results

**Fig 1:**
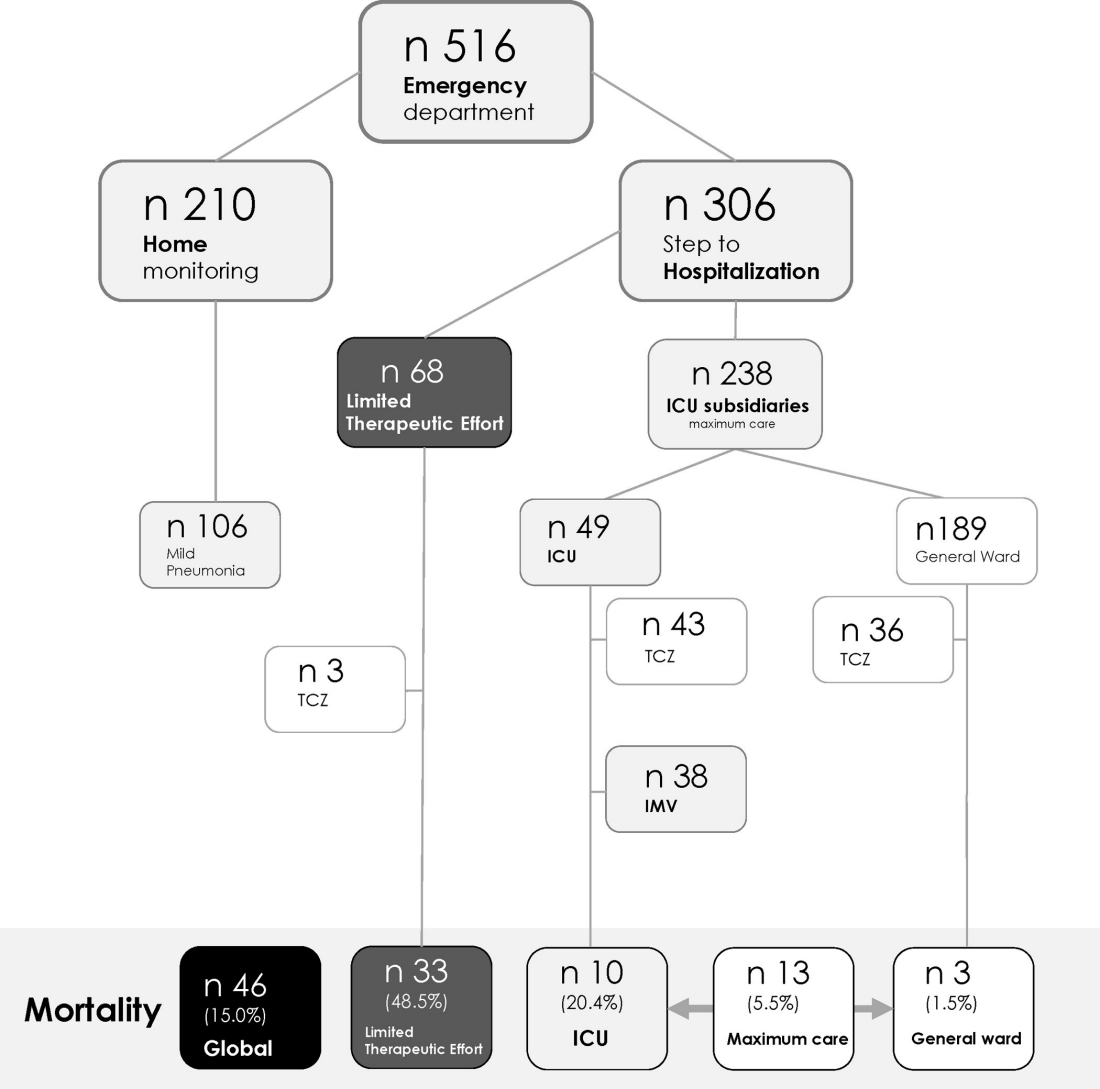
Flowchart of COVID-19 cases evaluated in the hospital. ICU: intensive care unit, TCZ: tocilizumab.

**Fig 2:**
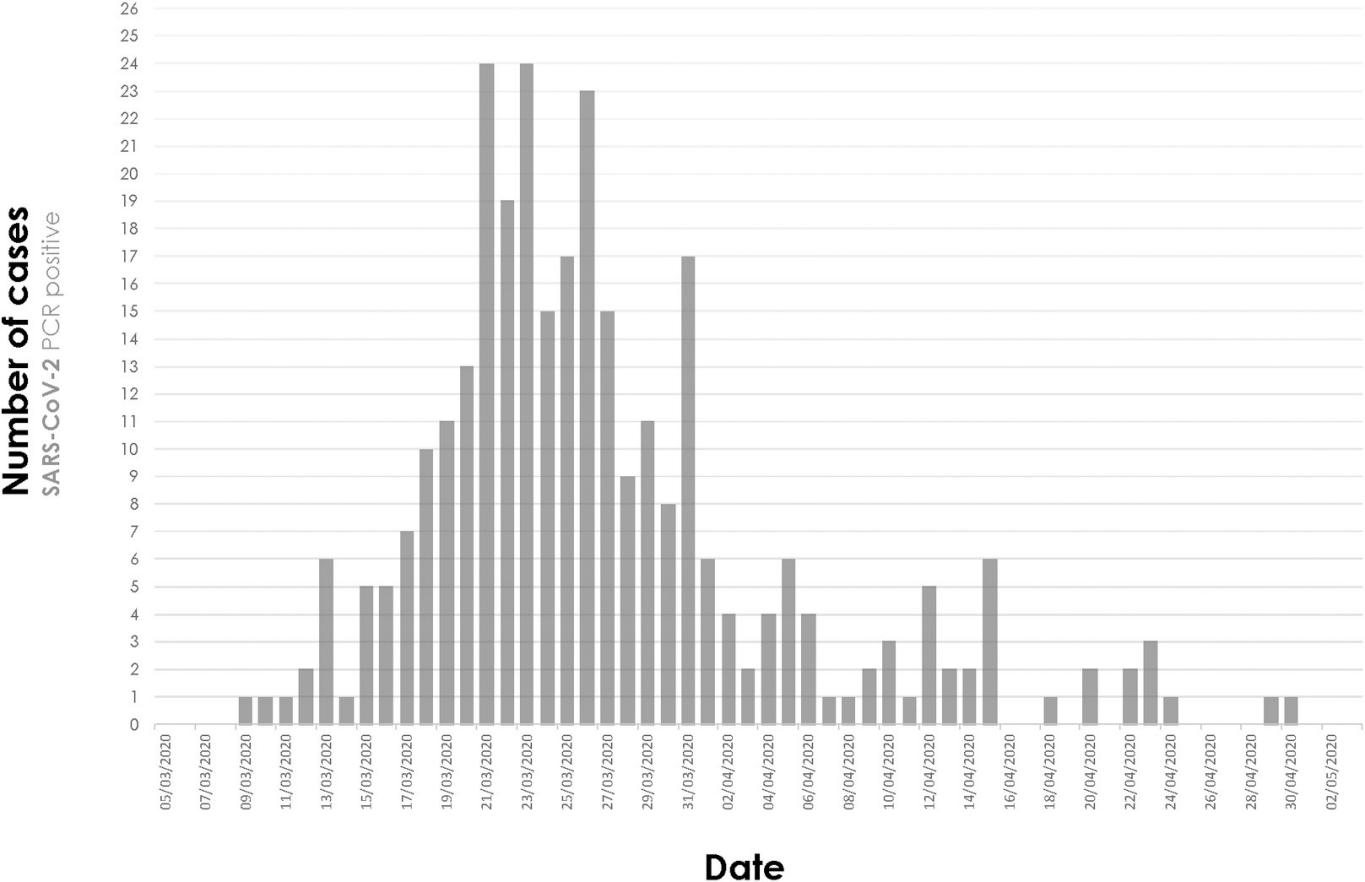
Epidemic curve of hospitalized patients in the health department during the study period (March 3 –May 2, 2020). Dates indicate the time of admission.

In the study period, 516 confirmed cases of COVID-19 were evaluated in the emergency department; 210 (106 with mild pneumonia) were managed as outpatients and 306 hospitalized (**Fig 1**). **Fig 2** shows the epidemic curve of hospitalized patients in the health department during the study period. At the time of analysis, 16 patients remained hospitalized (eight at ICU, seven under IMV). Seven readmissions occurred (2.3%), and no patient was lost to follow-up. Median (IQR) length of admission and follow-up were 9 (5–14) and 43 days (33–48), respectively.

The hospitalized population was mostly middle-aged with similar gender distribution, frequent comorbidities, raised inflammatory markers and lymphopenia, and one out of four with extensive lung radiological opacities [**Table 1**]. Caucasians were 91.8% of the cohort. SARS-CoV-2 was detected by RT-PCR in 289 patients (94.4%), in 17 (5.6%) COVID-19 diagnosis was clinical–eight of them later found positive for SARS-CoV-2 antibodies in the outpatient follow-up.

**Table 1:** General characteristics of the study population and comparison according to the management approach.

	*Total population [n = 306]*	*Maximum care [n = 238]*	*Limited therapeutic effort [n = 68]*	*p*
**Demographics**				
Age (median), years	65.0 (51.0–77.0)	60.5 (46.0–71.0)	87.0 (79.0–90.0)	**<0.001**
Age (quartiles), %				
• Q1 (<51.0)	23.9	30.3	1.5	**<0.001**
• Q2 (51.0–64.9)	25.8	32.4	2.9	
• Q3 (65.0–76.9)	24.8	27.7	14.7	
• Q4 (≥77.0)	25.5	9.7	80.9	
Males, %	57.8	59.7	51.5	.228
Nosocomial, %	5.6	3.4	13.2	**.004**
Long-term care resident, %	7.2	0.4	30.9	**<0.001**
Health professional, %	9.2	11.8	0.0	**.003**
**Comorbidities**				
Hypertension, %	49.2	40.9	77.9	**<0.001**
Diabetes, %	22.0	18.1	35.3	**.003**
Body mass index, kg/m2	27.4 (24.2–31.6)	27.5 (24.4–31.6)	26.7 (23.9–31.1)	.339
Obesity, %	34.3	34.7	32.7	.790
Cardiovascular disease, %	16.2	10.1	37.7	**<0.001**
Chronic respiratory disease, %	19.9	16.2	32.4	**.003**
Immunosuppression, %	6.9	8.4	1.5	.055
Charlson comorbidity index	3.0 (1.0–5.0)	2.0 (1.0–4.0)	7.0 (6.0–9.0)	**<0.001**
Charlson index ≥3, %	53.3	40.3	98.5	**<0.001**
10-years expected survival^a^	77.5 (21.0–95.9)	90.2 (53.4–95.9)	0.0 (0.0–2.3)	**<0.001**
**Clinical Presentation**				
Clinical duration, days^b^	6.0 (3.0–9.0)	7.0 (4.0–9.0)	3.0 (1.0–7.0)	**<0.001**
Fever, %	72.4	79.3	47.8	**<0.001**
Dry cough, %	61.1	66.1	43.3	**0.001**
Wet cough, %	17.8	17.3	19.4	.691
Dyspnea, %	53.6	51.3	61.8	.126
Diarrhoea, %	25.1	29.1	10.8	**.003**
Confusion, %	12.7	6.8	33.3	**<0.001**
Fatigue, %	40.2	46.3	18.8	**<0.001**
Myalgias-arthralgias, %	26.4	32.9	3.1	**<0.001**
Anosmia-dysgeusia, %	12.4	14.5	4.8	**.037**
**Initial Assessment**				
Oximetry at room air (%)	95.0 (92.0–97.0)	95.0 (93.0–97.0)	93.0 (90.0–96.0)	**.001**
PaO2:FiO2	329.0 (276.1–396.3)	338.0 (285.3–400.0)	302.5 (210.1–342.9)	**.002**
Respiratory rate, breaths/min	18.0 (16.0–24.0)	17.0 (16.0–23.8)	22.0 (16.0–28.3)	**.005**
Systolic BP, mmHg	130.0 (113.3–144.0)	130.0 (115.0–145.0)	126.5 (110.0–141.5)	.301
Diastolic BP, mmHg	77.0 (67.0–88.0)	80.0 (70.0–89.0)	67.0 (58.3–80.5)	**<0.001**
Heart rate, beats/min	94.5 (80.0–105.0)	96.0 (85.0–106.0)	85.0 (68.0–99.0)	**<0.001**
CURB65	1.0 (0.0–2.0)	1.0 (0.0–2.0)	3.0 (2.0–3.5)	**<0.001**
eGFR, ml/min/m^2^	81.0 (55.5–90.0)	87.0 (69.7–90.0)	37.3 (28.3–72.0)	**<0.001**
eGFR <60ml/min/m^2^, %	28.3	17.4	66.2	**<0.001**
Leukocytes, per mm^3^	6455.0 (5011.8–8800.0)	6292.0 (5000.0–8365.0)	6800.0 (5310.0–12940.0)	**.029**
Lymphocytes, per mm^3^	1040.0 (730.0–1390.0)	1040.0 (782.5–1377.5)	1040.0 (610.0–1492.5)	.531
C-reactive protein, mg/dL	6.1 (2.8–12.4)	5.5 (2.5–12.0)	9.0 (4.2–14.2)	**.032**
Procalcitonin, ng/mL	0.11 (0.06–0.20)	0.10 (0.05–0.18)	0.15 (0.09–0.41)	**<0.001**
Ferritin, mg/L	694.0 (330.0–1280.5)	690.0 (335.0–1286.0)	732.0 (284.5–1300.0)	.951
Lactate dehydrogenase, U/L	269.5 (220.0–368.0)	271.0 (220.0–369.0)	264.0 (226.0–374.5)	.633
D-dimers, mg/mL	0.64 (0.40–1.30)	0.60 (0.39–1.03)	1.21 (0.42–2.70)	**.004**
Interleukin 6, pg/mL	25.0 (10.0–59.0)	23.0 (10.0–55.0)	51.0 (15.0–94.0)	.085
Troponin T, ng/L	11.0 (6.0–22.5)	8.0 (5.0–16.0)	37.0 (20.0–55.0)	**<0.001**
Brain natriuretic peptide, pg/mL	143.0 (37.5–932.5)	75.5 (26.8–230.0)	2046.0 (581.0–4754.0)	**<0.001**
Creatine phosphokinase, U/L	78.5 (51.0–147.5)	85.0 (59.3–144.5)	64.0 (39.8–165.0)	.071
Aspartate aminotransferase, U/L	33.0 (23.0–53.0)	34.0 (24.0–53.0)	33.0 (22.0–54.0)	.806
Alanine aminotransferase, U/L	27.0 (16.0–44.0)	28.0 (17.0–46.0)	19.5 (13.0–36.3)	**.021**
Opacities >50% of lung surface on X-rays, %	23.1	19.5	36.2	**.008**

Data shown as % unless specified otherwise. In bold, statistically significant differences.

^a^10-years expected survival derived from Charlson comorbidity index score.

^b^Days of symptoms before admission. OR: odds ratio, 95%CI: 95% confidence interval.

Out of 306, 238 patients (77.8%) received maximum care and 68 (22.2%) LTE [**Table 1**]. Subgroups differed grossly in background and characteristics at presentation, with faster clinical deterioration after symptoms onset in the LTE group and a significant elevation of markers of cardiac involvement. Interestingly, inflammatory markers levels were similar.

In the maximum care group, management included hydroxychloroquine in 225 (94.5%): 32 (15.6%) as monotherapy, 134 (56.3%) plus azithromycin, 21 (8.8%) plus lopinavir-ritonavir, and 38 (16.0%) the three drugs. TCZ and corticosteroids were used in 79 (33.2%) and 74 patients (31.1%), respectively. Non-invasive respiratory support was initiated in 42 cases (17.6%)– 35 by high-flow nasal oxygen and seven by CPAP/BiPAP. Fifty-eight patients (85.3%) under LTE received hydroxychloroquine, combined with azithromycin in 34 (50.0%), three (4.4%) received triple therapy. TCZ was initiated in three patients (4.4%), and 18 (26.5%) received corticosteroids. Non-invasive respiratory support was supplied in nine cases (13.2%)—five by high-flow nasal oxygen and four by CPAP/BiPAP.

### Outcome: Fatality

Overall fatality rate was 15.0% (n = 46) (**Fig 1**). In the maximum care group, 13 patients (5.5%) died, ten at ICU and three at general wards. Six were considered directly COVID-19 related (five due to respiratory failure, one stroke); others were six ventilation-associated pneumonia and one difficult intubation-related cardiac arrest. Fatality rate in LTE was 48.5% (n = 33), 27 cases (81.8%) by COVID-19 and 6 (18.2%) due to concurrent infections.

**Fig 3:**
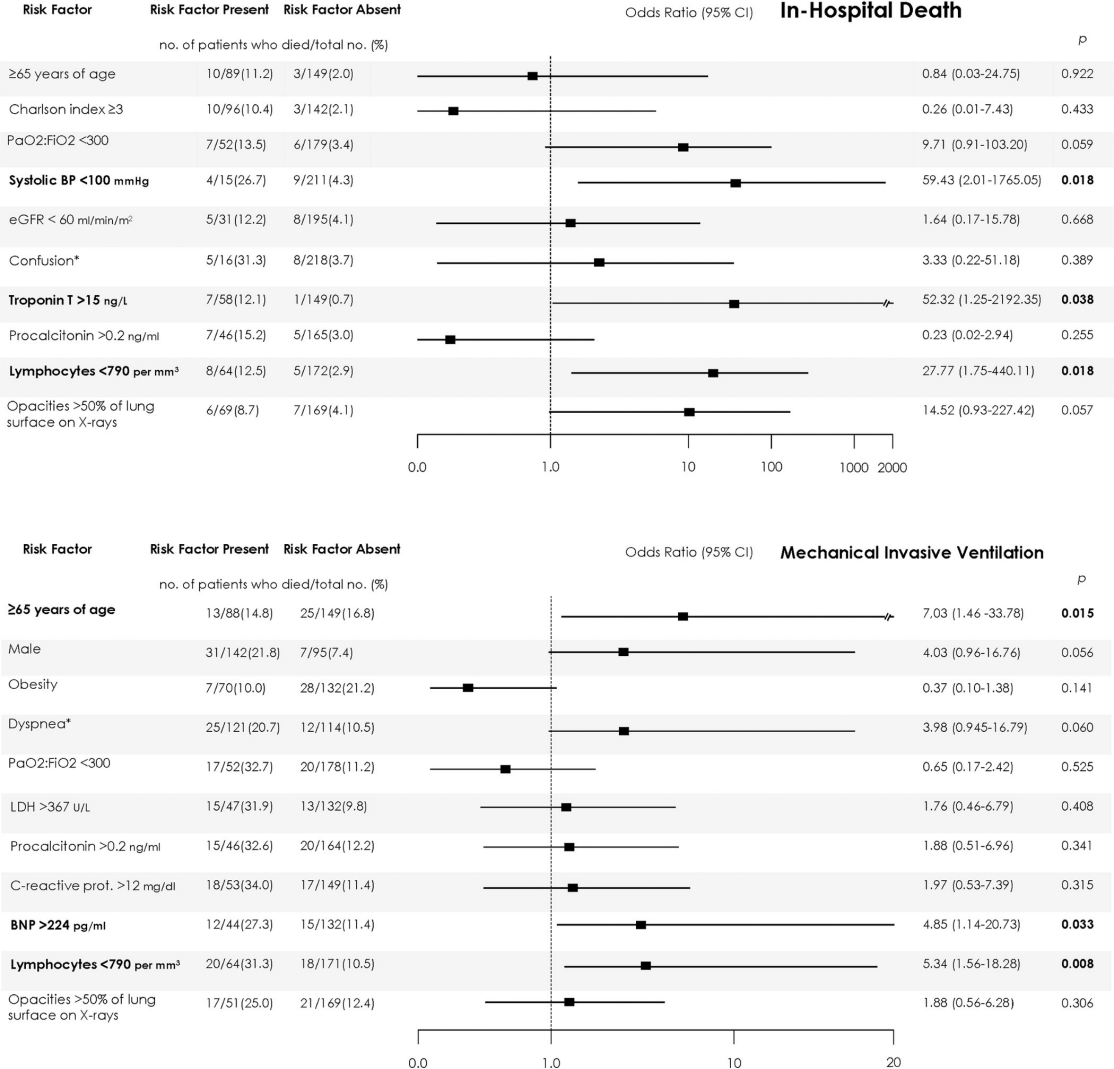
Independent risk factors of death (A) and invasive mechanical ventilation (B) in the maximum care population. Numbers and percentages of patients with each risk factor who had the outcomes (risk factor present) and of patients without each risk factor with favorable evolution (risk factor absent) are shown. The 95% confidence intervals (CIs) of the odds ratios have been adjusted for multiple testing. R^2^ of models: 0.55 for mortality, 0.45 for invasive mechanical ventilation. In bold, independent predictors associated with the outcomes. BP: blood pressure; eGFR: estimated glomerular filtration rate (by CKD-EPI formula); *on admission; LDH: lactate dehydrogenase; prot: protein; BNP: Brain natriuretic peptides. Multivariate models included 184 and 186 participants, respectively. A comparison between global population and population with complete data for the included covariates is provided in the **S2 Table**.

Significant associations between fatality and several explanatory variables were identified in the maximum care subgroup through the univariate regression models [**Table 2**]. After adjustment **(Fig 3)**, baseline independent risk factors of mortality were lymphocyte count <790/mm^3^ (OR 27.8; 95%CI 1.8–440.1), troponin T >15ng/L (OR 52.3; 95%CI 1.3–2192.4) and systolic blood pressure <100mmHg (OR 59.4; 95%CI 2.0–1765.1). PaO2:FiO2 ratio <300mmHg and having extended pulmonary opacities showed a trend towards significance.

**Table 2:** Fatality and risk factors in patients under maximum care.

	*Death (n = 13)*	*Unadjusted OR (95%CI)*	*p*
**Demographics**			
Age			
• <65 years	3/149 (2.0)	1.00 (ref)	-
• ≥65 years	10/89 (11.2)	6.16 (1.65–23.04)	**.007**
Gender			
• Females	3/95 (3.2)	1.00 (ref)	-
• Males	10/143 (7.0)	2.35 (0.63–8.77)	.204
Nosocomial case			
• No	11/229 (4.8)	1.00 (ref)	**-**
• Yes	2/8 (25.0)	6.61 (1.19–36.56)	**.031**
Long-term care resident			
• No	13/237 (5.5)	NC	-
• Yes	0/1 (0.0)		
Health professional			
• No	13/210 (6.2)	NC	-
• Yes	0/28 (0.0)		
**Comorbidities**			
Hypertension			
• No	6/139 (4.3)	1.00 (ref)	**-**
• Yes	7/98 (7.1)	1.71 (0.56–5.24)	.351
Diabetes			
• No	8/194 (4.1)	1.00 (ref)	**-**
• Yes	5/43 (11.6)	3.08 (0.95–9.92)	.060
Obesity			
• No	6/132 (4.5)	1.00 (ref)	**-**
• Yes	6/70 (8.6)	0.51 (0.16–1.64)	.257
Cardiovascular disease			
• No	10/218 (4.6)	1.00 (ref)	**-**
• Yes	3/19 (15.8)	4.37 (1.03–18.55)	**.046**
Chronic respiratory disease			
• No	10/196 (5.1)	1.00 (ref)	**-**
• Yes	3/38 (7.9)	1.60 (0.42–6.12)	.490
Immunosuppression			
• No	13/217 (6.0)	1.00 (ref)	**-**
• Yes	0/20 (0.0)	NC	-
Charlson index			
• <3	3/142 (2.1)	1.00 (ref)	**-**
• ≥3	10/96 (10.4)	5.39 (1.44–20.13)	**.012**
10-years expected survival^a^			
• ≥90%	3/122 (2.5)	1.00 (ref)	**-**
• <90%	7/81 (8.6)	3.75 (0.94–14.96)	.061
**Clinical Presentation**			
Clinical duration^b^			
• ≥7 days	4/110 (3.6)	1.00 (ref)	**-**
• <7 days	6/88 (6.8)	1.94 (0.53–7.10)	.317
Fever			
• No	1/49 (2.0)	1.00 (ref)	**-**
• Yes	11/188 (5.9)	2.98 (0.38–23.68)	.301
Dry cough			
• No	2/80 (2.5)	1.00 (ref)	**-**
• Yes	10/156 (6.4)	2.67 (0.57–12.50)	.212
Wet cough			
• No	10/196 (5.1)	1.00 (ref)	**-**
• Yes	2/41 (4.9)	0.95 (0.20–4.53)	.953
Dyspnea			
• No	3/115 (2.6)	1.00 (ref)	**-**
• Yes	10/121 (8.3)	3.36 (0.90–12.55)	.071
Diarrhoea			
• No	8/166 (4.8)	1.00 (ref)	**-**
• Yes	4/68 (5.9)	1.23 (0.36–4.24)	.738
Confusion			
• No	8/218 (3.7)	1.00 (ref)	**-**
• Yes	5/16 (31.3)	11.93 (3.35–42.54)	**<0.001**
Fatigue			
• No	5/122 (4.1)	1.00 (ref)	**-**
• Yes	7/105 (6.7)	1.67 (0.51–5.43)	.393
Myalgias-arthralgias			
• No	10/155 (6.5)	1.00 (ref)	**-**
• Yes	3/76 (3.9)	0.60 (0.16–2.23)	.442
Anosmia-dysgeusia			
• No	11/194 (5.7)	1.00 (ref)	**-**
• Yes	1/33 (3.0)	0.52 (0.07–4.17)	.538
**Initial Assessment**			
Oximetry at room air			
• ≥94%	2/132 (1.5)	1.00 (ref)	**-**
• <94%	11/95 (11.6)	8.51 (1.84–39.37)	**.006**
PaO2:FiO2			
• ≥300	6/179 (3.4)	1.00 (ref)	**-**
• <300	7/52 (13.5)	7.40 (1.44–38.05)	**.017**
Respiratory rate			
• ≤24 breaths/min	6/110 (5.5)	1.00 (ref)	**-**
• >24 breaths/min	2/18 (11.1)	1.88 (0.42–8.36)	.400
Systolic BP			
• ≥100 mmHg	9/211 (4.3)	1.00 (ref)	**-**
• <100 mmHg	4/15 (26.7)	8.08 (2.15–30.40)	**.002**
Diastolic BP			
• ≥60 mmHg	10/204 (4.9)	1.00 (ref)	**-**
• <60 mmHg	3/22 (13.6)	3.06 (0.78–12.10)	.110
Heart rate			
• ≤100 beats/min	8/151 (5.3)	1.00 (ref)	**-**
• >100 beats/min	5/80 (6.3)	1.19 (0.38–3.77)	.765
CURB65			
• <3	3/114 (2.6)	1.00 (ref)	**-**
• ≥3	5/10 (50.0)	37.00 (6.84–200.26)	**<0.001**
eGFR			
• ≥60 mL/min/m^2^	8/195 (4.1)	1.00 (ref)	**-**
• <60 mL/min/m^2^	5/41 (12.2)	3.25 (1.01–10.49)	**.049**
Leukocytes			
• ≤8300 per mm^3^	7/150 (4.7)	1.00 (ref)	**-**
• >8300 per mm^3^	3/52 (5.8)	1.25 (0.31–5.03)	.753
Lymphocytes			
• ≥790 per mm^3^	5/172 (2.9)	1.00 (ref)	**-**
• <790 per mm^3^	8/64 (12.5)	4.47 (1.21–16.51)	**.025**
C-reactive protein			
• ≤12 mg/dL	5/149 (3.4)	1.00 (ref)	**-**
• >12 mg/dL	5/53 (9.4)	3.00 (0.83–10.81)	.093
Procalcitonin			
• ≤0.2 ng/mL	5/165 (3.0)	1.00 (ref)	**-**
• >0.2 ng/mL	7/46 (15.2)	5.74 (1.73–19.07)	**.004**
Ferritin			
• ≤1300 mg/L	4/136 (2.9)	1.00 (ref)	**-**
• >1300 mg/L	1/45 (2.2)	0.75 (0.08–6.89)	.799
Lactate dehydrogenase			
• ≤367 U/L	4/132 (3.0)	1.00 (ref)	**-**
• >367 U/L	3/47 (6.4)	2.18 (0.47–10.13)	.319
D-dimers			
• ≤1 mg/mL	5/137 (3.6)	1.00 (ref)	**-**
• >1 mg/mL	3/45 (6.7)	1.89 (0.43–8.23)	.399
Interleukin 6			
• ≤54 pg/mL	2/114 (1.8)	1.00 (ref)	**-**
• >54 pg/mL	2/38 (5.3)	3.11 (0.42–22.89)	.265
Troponin T			
• ≤15 ng/L	1/149 (0.7)	1.00 (ref)	**-**
• >15 ng/L	7/58 (12.1)	15.73 (1.79–138.56)	**.013**
Brain natriuretic peptide			
• ≤224 pg/mL	3/132 (2.3)	1.00 (ref)	**-**
• >224 pg/mL	3/44 (6.8)	3.15 (0.61–16.19)	.170
Creatine phosphokinase			
• ≤146 U/L	8/140 (5.7)	1.00 (ref)	**-**
• >146 U/L	1/48 (2.1)	0.35 (0.04–2.88)	.330
Aspartate aminotransferase			
• ≤54 U/L	5/146 (3.4)	1.00 (ref)	**-**
• >54 U/L	3/50 (6.0)	1.80 (0.41–7.82)	.433
Alanine aminotransferase			
• ≤44 U/L	5/138 (3.6)	1.00 (ref)	**-**
• >44 U/L	3/58 (5.2)	1.45 (0.34–6.28)	.619
Opacities of lung surface on X-rays	≥		
• ≤50%	7/169 (4.1)	1.00 (ref)	**-**
• >50%	6/69 (8.7)	3.97 (1.26–12.53)	**.019**

Data shown as n (%) unless specified otherwise. In bold, statistically significant differences.

^a^10-years expected survival derived from Charlson comorbidity index score.

^b^Days of symptoms before admission. OR: odds ratio, 95%CI: 95% confidence interval, NC: not calculable.

The association analyses for the LTE group can be found in **S1 Table**. Median Charlson index of those who died was 8.0, while was 6.0 in survivors. Significant differences in PaO2:FiO2 <300mmHg and several laboratory variables were also noted. Multivariate regression was not possible as the low number of patients precluded such an analysis, given the instability of the model.

### Outcome: Invasive mechanical ventilation

Forty-nine patients (20.6%) under maximum care were admitted at ICU, median stay nine days (6–16); 38 (16.0%) required IMV, half in the first 48h since admission, lasting a median of 8.5 days (6.0–14.5). One patient required ECMO support. Seven patients developed ventilation-associated pneumonia (VAP), incidence 13.1 per 1.000 days of intubation.

Significant associations between the use of IMV and several explanatory variables were identified [**Table 3**]. After adjustment (**Fig 3**), advanced age (OR 7.0; 95CI 1.5–33.8), lymphocyte counts <790/mm^3^ (OR 5.3; 95%CI 1.6–18.3) and brain natriuretic peptides (BNP) >240pg/mL (OR 4.9; 95%CI 1.1–20.7) at presentation were independently associated with requiring IMV during the admission. Being male showed a trend toward significance.

**Table 3:** Requirement and risk factors of invasive mechanical ventilation in patients under maximum care.

	*IMV (n = 38)*	*Unadjusted OR (95%CI)*	*p*
**Demographics**			
Age			
• <65 years	25/149 (16.8)	1.00 (ref)	-
• ≥65 years	13/88 (14.8)	0.85 (0.41–1.76)	.658
Gender			
• Females	7/95 (7.4)	1.00 (ref)	**-**
• Males	31/142 (21.8)	3.55 (1.49–8.44)	**.004**
Nosocomial case			
• No	35/228 (15.4)	1.00 (ref)	**-**
• Yes	2/8 (25.0)	1.85 (0.36–9.53)	.463
Long-term care resident			
• No	38/236 (16.1)	NC	-
• Yes	0/1 (0.0)		
Health professional			
• No	35/209 (16.7)	1.00 (ref)	**-**
• Yes	3/28 (10.7)	0.60 (0.17–2.10)	.424
**Comorbidities**			
Hypertension			
• No	20/139 (14.4)	1.00 (ref)	**-**
• Yes	18/97 (18.6)	1.34 (0.67–2.69)	.412
Diabetes			
• No	30/193 (15.5)	1.00 (ref)	**-**
• Yes	8/43 (18.6)	1.26 (0.53–2.97)	.602
Obesity			
• No	28/132 (21.2)	1.00 (ref)	**-**
• Yes	7/70 (10.0)	0.41 (0.17–1.00)	**.050**
Cardiovascular disease			
• No	35/218 (16.1)	1.00 (ref)	**-**
• Yes	3/18 (16.7)	0.99 (0.27–3.64)	.992
Chronic respiratory disease			
• No	34/195 (17.4)	1.00 (ref)	**-**
• Yes	4/38 (10.5)	0.56 (0.19–1.69)	.308
Immunosuppression			
• No	36/216 (16.7)	1.00 (ref)	**-**
• Yes	2/20 (10.0)	0.56 (0.12–2.51)	.448
Charlson index			
• <3	23/142 (16.2)	1.00 (ref)	**-**
• ≥3	15/95 (15.8)	0.96 (0.47–1.95)	.906
10-years expected survival^a^			
• ≥90%	21/122 (17.2)	1.00 (ref)	**-**
• <90%	14/81 (17.3)	1.01 (0.48–2.11)	.990
**Clinical Presentation**			
Clinical duration^b^			
• ≥7 days	18/110 (16.4)	1.00 (ref)	**-**
• <7 days	17/88 (19.3)	1.22 (0.58–2.54)	.588
Fever			
• No	2/48 (4.2)	1.00 (ref)	**-**
• Yes	36/188 (19.1)	5.57 (1.29–23.99)	.021
Dry cough			
• No	8/80 (10.0)	1.00 (ref)	**-**
• Yes	29/155 (18.7)	2.06 (0.89–4.73)	.091
Wet cough			
• No	31/195 (15.9)	1.00 (ref)	**-**
• Yes	7/41 (17.1)	1.10 (0.45–2.69)	.824
Dyspnea			
• No	12/114 (10.5)	1.00 (ref)	**-**
• Yes	25/121 (20.7)	2.24 (1.06–4.70)	**.034**
Diarrhoea			
• No	27/165 (16.4)	1.00 (ref)	**-**
• Yes	11/68 (16.2)	0.99 (0.46–2.14)	.987
Confusion			
• No	32/217 (14.7)	1.00 (ref)	**-**
• Yes	5/16 (31.3)	2.64 (0.86–8.11)	.090
Fatigue			
• No	15/121 (12.4)	1.00 (ref)	**-**
• Yes	22/105 (21.0)	1.89 (0.92–3.87)	.081
Myalgias-arthralgias			
• No	25/154 (16.2)	1.00 (ref)	**-**
• Yes	12/76 (15.8)	0.98 (0.46–2.07)	.947
Anosmia-dysgeusia			
• No	34/193 (17.6)	1.00 (ref)	**-**
• Yes	3/33 (9.1)	0.47 (0.14–1.63)	.235
**Initial Assessment**			
Oximetry at room air			
• ≥94%	8/131 (6.1)	1.00 (ref)	**-**
• <94%	28/95 (29.5)	6.48 (2.80–15.01)	**<0.001**
PaO2:FiO2			
• ≥300	12/113 (10.6)	1.00 (ref)	**-**
• <300	16/51 (31.4)	3.85 (1.66–8.93)	**.002**
Respiratory rate			
• ≤24 breaths/min	12/110 (10.9)	1.00 (ref)	**-**
• >24 breaths/min	8/18 (44.4)	16.06 (5.15–50.05)	**<0.001**
Systolic BP			
• ≥100 mmHg	32/210 (15.2)	1.00 (ref)	**-**
• <100 mmHg	4/15 (26.7)	0.50 (0.15–1.66)	.256
Diastolic BP			
• ≥60 mmHg	32/203 (15.8)	1.00 (ref)	**-**
• <60 mmHg	4/22 (18.2)	0.84 (0.27–2.64)	.761
Heart rate			
• ≤100 beats/min	25/150 (16.7)	1.00 (ref)	**-**
• >100 beats/min	10/80 (12.5)	0.72 (0.33–1.59)	.415
CURB65			
• <3	13/114 (11.4)	1.00 (ref)	**-**
• ≥3	5/10 (50.0)	7.77 (1.98–30.50)	**.003**
eGFR			
• ≥60 mL/min/m^2^	29/194 (14.9)	1.00 (ref)	**-**
• <60 mL/min/m^2^	7/41 (17.1)	1.18 (0.48–2.91)	.722
Leukocytes			
• ≤8300 per mm^3^	29/150 (19.3)	1.00 (ref)	**-**
• >8300 per mm^3^	6/52 (11.5)	0.54 (0.21–1.40)	.206
Lymphocytes			
• ≥790 per mm^3^	18/171 (10.5)	1.00 (ref)	**-**
• <790 per mm^3^	20/64 (31.3)	4.45 (2.07–9.53)	**<0.001**
C-reactive protein			
• ≤12 mg/dL	17/149 (11.4)	1.00 (ref)	**-**
• >12 mg/dL	18/53 (34.0)	3.99 (1.87–8.54)	**<0.001**
Procalcitonin			
• ≤0.2 ng/mL	20/164 (12.2)	1.00 (ref)	**-**
• >0.2 ng/mL	15/46 (32.6)	3.51 (1.62–7.61)	**.001**
Ferritin			
• ≤1300 mg/L	15/136 (11.0)	1.00 (ref)	**-**
• >1300 mg/L	15/45 (33.3)	4.03 (1.78–9.16)	**.001**
Lactate dehydrogenase			
• ≤367 U/L	13/132 (9.8)	1.00 (ref)	**-**
• >367 U/L	15/47 (31.9)	4.29 (1.85–9.93)	**.001**
D-dimers			
• ≤1 mg/mL	17/137 (12.4)	1.00 (ref)	**-**
• >1 mg/mL	10/45 (22.2)	2.02 (0.85–4.80)	.113
Interleukin 6			
• ≤54 pg/mL	9/114 (7.9)	1.00 (ref)	**-**
• >54 pg/mL	12/38 (31.6)	5.39 (2.05–14.13)	**.001**
Troponin T			
• ≤15 ng/L	20/148 (13.5)	1.00 (ref)	**-**
• >15 ng/L	12/58 (20.7)	1.96 (0.84–4.53)	.118
Brain natriuretic peptide			
• ≤224 pg/mL	15/132 (11.4)	1.00 (ref)	**-**
• >224 pg/mL	12/44 (27.3)	2.93 (1.25–6.87)	**.014**
Creatine phosphokinase			
• ≤146 U/L	19/140 (13.6)	1.00 (ref)	**-**
• >146 U/L	15/48 (31.3)	2.90 (1.33–6.31)	**.007**
Aspartate aminotransferase			
• ≤54 U/L	18/146 (12.3)	1.00 (ref)	**-**
• >54 U/L	16/50 (32.0)	3.35 (1.55–7.24)	**.002**
Alanine aminotransferase			
• ≤44 U/L	19/138 (13.8)	1.00 (ref)	**-**
• >44 U/L	15/58 (25.9)	2.19 (1.02–4.68)	**.044**
Opacities of lung surface on X-rays			
• ≤50%	21/169 (12.4)	1.00 (ref)	**-**
• >50%	17/68 (25.0)	4.51 (2.08–9.80)	**<0.001**

Data shown as n (%) unless specified otherwise. In bold, statistically significant differences.

^a^10-years expected survival derived from Charlson comorbidity index score.

^b^Days of symptoms before admission. OR: odds ratio, 95%CI: 95% confidence interval, IMV: invasive mechanical ventilation, NC: not calculable.

## Discussion

The present cohort is characterized by the homogeneous management and outcomes interpretation regarding patients’ life expectancies and comorbidities by a multi-disciplinary team. Overall fatality rate was 15.0%, 5.5% for patients with maximum care (among whom 16% needed intubation) and 48.5% in those with LTE. These results suggest that published fatality rates, widely variable (1.4–28.3%), does not capture the different included populations. Median censoring was 43 days, and only 5% of patients remained hospitalized at the time of analysis. Major biases, when comparing different series, relate to 1) study population, 2) patients’ characteristics and consequent care, 3) disease severity, 4) time of follow-up and data completeness. It seems inappropriate to compare crude fatality rates without considering these factors.

Diagnostic testing at the population level will capture mild and asymptomatic cases and influence outcome assessment. Case fatality rate will be overestimated if only severe cases are considered [31]. Indication of testing in the few published series at population level is unclear. In these reports, mortality was 2% in China [10], 2% in California [32] and 7% in Italy [20]. In Spain, the shortage in microbiological tests impeded population screening, so overall mortality is unknown. In the present cohort, testing was systemically performed only in patients with moderate or severe respiratory infection attending the Emergency Department (ED); out of 510 patients seen at ED, 46 died (9.0%). In a recent series from Madrid [33], 14% of patients assessed at ED died. Most studies focus on hospitalized patients, with percentages of fatality being 1–28% in China [5, 7, 9, 11, 16], 10–21% in USA [34, 35], and 21% in Madrid [36]. Here, the overall fatality was 15%.

The analysis of fatality in COVID-19 should consider the disease severity to compare across population and to identify outcome predictors [37]. In the absence of validated scales for COVID-19, a reasonable approach is to use the fatality/IMV ratio to compare severity. It provides a more objective picture of each study population, the results have been diverse: Chen et al. 2.75 (11.0/4.0) [16], Madrid data 1.95 (20.7/10.6) [36], Richardson et al. 1.72 (21.0/12.2) [34], Zhou et al. 1.69 (28.3/16.7) [5], Wang et al. 0.92 (4.3/12.3) [17], Guan et al. 0.61 (1.4/2.3) [4], Myers et al. 0.53 (15.6/29.2) [32], Liu et al. 0.46 (11.7/25.0) [9] and Goyal et al. 0.31 (10.2/33.0) [35]. The fatality/IMV ratio in the present cohort was 1.20 (15.0/12.5). This estimation permits a global view of fatality according to severity. The high number of patients still intubated in most series indeed underestimated mortality. This approach is however imperfect, as is affected by patients’ background and available resources.

**Fig 4:**
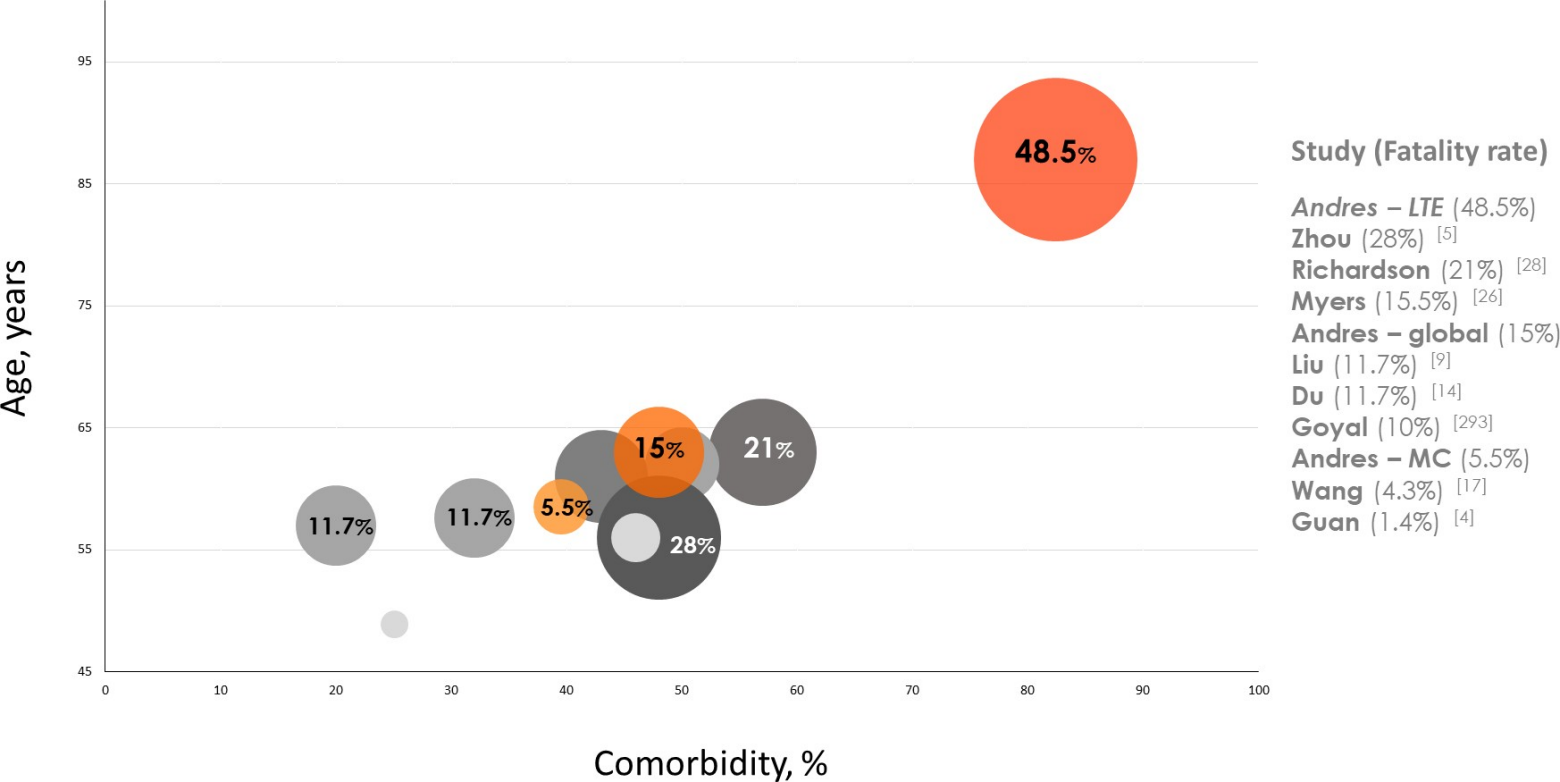
Age and comorbidity—adjusted distribution of fatality among reported hospitalized series with >100 patients and Alicante cohort, stratified according to the management approach. Size of circles represent the magnitude of fatality rate for each series. LTE: limited therapeutic effort, MC: maximum care.

In the literature, there are substantial variations in fatality numbers across studies, considering patients’ background [**Fig 4**]. Chinese populations tend to be younger and with less comorbidity than Westerns. Patients’ background heavily conditions treatment decisions during admission, so stratifying on the therapeutic effort facilitates the analysis of COVID-19 fatality (**Fig 4**). The maximum care, despite middle age, high comorbidity (40% Charlson index ≥3) and moderate-severe pneumonia (20% admitted to ICU), had successful outcomes with low fatality (5.5%). Conversely, in the population with LTE, numbers dramatically raised, accounting for almost three of every four deaths. Rate was likely ameliorated by the extensive use of antivirals, glucocorticoids and non-invasive respiratory support, as well as the management of concurrent comorbid decompensations. The decided care, in a population with median 87 years of age and estimated 10-years survival of 0%, is in keeping with management of other infectious diseases in similar patients. This stratified analysis, not previously covered in the COVID-19 literature, contributes to understand the disease outcome according to the patients’ characteristics.

Differences in follow-up time and proportions of patients who remain hospitalized may bias fatality statistics and limit the validity of some series, as authors acknowledged [34]. Here, follow-up was 43 days while, in most of the studies, this point is not provided, or data are truncated at discharge. About 50–60% of patients remain hospitalized in series from China [7, 17]. At the ICU setting, rates are even higher (58–72% of all intubated cases [21, 34]). In the current report, only 5% of patients remained hospitalized at the time of analysis (2.6% at ICU).

Pressure on hospital resources and shortage of essential equipment such as ventilators can also affect COVID-19 outcomes [38, 39]. In the present cohort, in-advanced arrangement of resources, organization under multi-disciplinary teams led by experts in infectious diseases and pneumology, and daily meetings to discuss complex cases and agreed decisions, provided a safe, supportive environment for patients and physicians facing COVID-19.

Having risk prediction tools is crucial when facing COVID-19 patients within a pandemic with limited health resources. Nevertheless, predictors should also be assessed considering the different clinical scenarios. Patients under LTE showed shorter time to admission, less fever, more confusion, a more inflammatory and prothrombotic laboratory profile, and more extensive lung involvement. Evaluating prognostic factors without considering these differences may result inaccurate. Under maximum care, mortality was associated with lower lymphocyte counts, hypotension and raised troponin T. Lymphopenia, older age and raised BNP predicted IMV during admission.

To date, only two series have found independent prognostic factors using multivariate analyses. Zhou et al. [5] in a retrospective analysis of 171 patients (28% mortality) identified age, SOFA and D-dimers. Du et al. [11], in a prospective study of 179 patients (12% mortality), found that age, established cardiovascular disease, CD3^+^CD8^+^ T-cell depletion and troponins were associated with increased mortality. The present identification of lymphopenia and troponin T supports former findings.

The grim prognosis of lymphopenia in COVID-19 seems firmly established. Low lymphocyte count (here <790/mm^3^) has been confirmed as independent predictor of mortality and need of IMV. The neutrophil/lymphocyte ratio, an attempt to standardize the total lymphocyte count, also determined mortality [40, 41]. T cells (CD3, CD4, CD8) levels decrease in severe disease [42], potentially due to direct invasion by SARS-CoV2, viral-induced autoimmune antibodies or apoptosis activation by proinflammatory cytokines (TNFα, IL-4). Hypercytokemia may also induce T-cell dysfunction [42]. More research is needed regarding pathophysiology of lymphopenia in COVID-19, but cumulative data suggest that profound lymphopenia (absolute or relative to neutrophils, or subpopulations of T lymphocytes) at presentation should be taken as a serious marker and, eventually, lead to intensifying vigilance and treatment.

The role of heart disease in COVID-19 mortality is an area of evolving research. Hypotension and raised BNP and troponins at presentation strongly suggest heart involvement. The associations with subsequent disease worsening [37] and fatal outcome ([11], present report) support the role of troponins as a useful marker of disease progression and prognosis in COVID-19. Elevated troponin levels are frequent in COVID-19, ranging 12–28% patients [5, 15, 18, 43], this rise correlates with ICU admission [18] and in-hospital mortality [5, 18, 43, 44]. Several mechanisms may explain the myocardial damage: direct viral invasion of cardiomyocytes (viral myocarditis), reduced oxygen supply, severe lung failure, microangiopathy-endothelial dysfunction, and SARS-CoV-2-derived cytokine storm [45, 46]. Besides, it might be attributable to the decreased activity of angiotensin-converting enzyme 2 (ACE2) receptors in the heart [47]. SARS-CoV-2 seems to infect host cells through ACE2 [47, 48], promoting ACE2 depletion and an imbalance of the renin-angiotensin-aldosterone and ACE2/angiotensin 1–7 axes with marked elevations of deleterious angiotensin-II levels, promoting vasoconstriction and proinflammatory, profibrotic effects. Nonetheless, direct evidence demonstrating that SARS-CoV-2 infects the human heart and decreases the ACE2 expression is currently lacking. Present data add more evidence to probable cardiovascular involvement at early stages of COVID-19. Monitoring myocardial enzymes as troponins, at the time of hospital admission, could help for risk stratification and potentially lead to earlier and more intensive therapy.

Level of D-dimers determined at ED, previously identified as an independent factor [5], has not been confirmed neither in the series by Du et al. [14] or in the present report. The lack of associations with other variables such as ferritin or lactate dehydrogenase is not surprising, due to the stratified clinical management and the comprehensive statistical analysis to rule out confounders.

The present study adds more evidence to some factors, especially lymphopenia and troponins, which should be included in risk assessment tools. Recently, Liang and colleagues have validated a clinical risk score (COVID-GRAM) to predict the occurrence of critical illness in COVID-19 inpatients [49]. According to previous considerations, this tool was developed in an Asian population with a less severe disease (<10% being critical) and consequently needs replication.

As limitations, this is an observational, retrospective, single-center study, and collection of data was not systematized in advance. Efforts were undertaken to capture and revise data by a clinical team with experience in COVID-19. The categorization and care approach were decided on clinical grounds based on patients’ characteristics but following no standardized criterion. However, subgroups were markedly different except for typical COVID-19 laboratory findings of inflammation. Long follow-up and no losses reinforce the present data. Nine cases were included despite negative microbiological testing as patients had the characteristic COVID-19 picture, as supported by the literature [50]; nevertheless, the potential influence on the study results seems very low. SOFA score was applied only at ICU admission and could not be evaluated as a prognostic factor [5]. The small sample size impeded valid multivariate analysis in LTE group and accounts for the wide 95%CI intervals obtained in maximum care. The impact of treatments was not studied as hampered by the sample size; it would require dividing on the care approach, severity (a rapidly evolving disease would require aggressive management more often) and other factors; this issue should be answered by larger, multi-center cohorts and controlled trials. As this is a hospital-based cohort, predictors might not be applicable to outpatients with COVID-19.

## Conclusion

This hospital-based cohort from Spain shows the outcome of 306 patients with COVID-19, managed by a multi-disciplinary unit with a shared protocol. Overall fatality was 15%, but markedly differed regarding the decided management approach, steadily differentiating two clinical scenarios, which translates in fatality rates nine-fold higher. Following a maximum care (immunomodulation, invasive ventilation), risk factors at presentation of subsequent mortality were lymphopenia, hypotension and high troponin T. Comorbidities led mortality in patients on LTE. These findings contribute to better define the COVID-19 picture and lay the groundwork for reporting fatality rates in future research.

## Supporting information

S1 Table Fatality and risk factors in patients managed under limited therapeutic effort. (DOCX)

S2 Table Results of simple regression models for fatality, for whole population and for population with complete data in the final multiple model. (DOCX)
