# Supplementary material for: Fatality and risk features for prognosis in COVID-19 according to the care approach – a retrospective cohort study
Source: PLoS One. 2021 Mar 23;16(3):e0248869. doi: 10.1371/journal.pone.0248869 (PMC7987197; doi:10.1371/journal.pone.0248869)
Supplement: S1 Table — (DOCX) [file pone.0248869.s001.docx]

**S1 Table. Fatality and risk factors in patients managed under limited therapeutic effort.**

|  | *Death* | *OR (95%CI)* | *p* |
| --- | --- | --- | --- |
| **Demographics** |  |  |  |
| Age |  |  |  |
| - <65 years | 1/3 (33.3) | 1.00 (ref) | - |
| - ≥65 years | 32/65 (49.2) | 1.94 (0.17-22.46) | .596 |
| Gender |  |  |  |
| - Females | 14/33 (42.4) | 1.00 (ref) | - |
| - Males | 19/35 (54.3) | 1.61 (0.62-4.20) | .329 |
| Nosocomial case |  |  |  |
| - No | 27/59 (45.8) | 1.00 (ref) | - |
| - Yes | 6/9 (66.7) | 2.37 (0.54-10.39) | .252 |
| Long-term care resident |  |  |  |
| - No | 22/47 (46.8) | 1.00 (ref) | - |
| - Yes | 11/21 (52.4) | 1.25 (0.45-3.50) | .671 |
| Health professional |  |  |  |
| - No | 33/68 (48.5) | NC | - |
| - Yes | 0/0 (0.0) |  |  |
| **Comorbidities** |  |  |  |
| Hypertension |  |  |  |
| - No | 5/15 (33.3) | 1.00 (ref) | - |
| - Yes | 28/53 (52.8) | 2.24 (0.67-7.45) | .188 |
| Diabetes |  |  |  |
| - No | 17/44 (38.6) | 1.00 (ref) | - |
| - Yes | 16/24 (66.7) | 3.18 (1.12-9.02) | **.030** |
| Obesity |  |  |  |
| - No | 16/35 (45.7) | 1.00 (ref) | - |
| - Yes | 7/17 (41.2) | 1.20 (0.37-3.89) | .757 |
| Cardiovascular disease |  |  |  |
| - No | 23/48 (47.9) | 1.00 (ref) | - |
| - Yes | 10/20 (50.0) | 0.83 (0.27-2.54) | .748 |
| Chronic respiratory disease |  |  |  |
| - No | 20/46 (43.5) | 1.00 (ref) | - |
| - Yes | 13/22 (59.1) | 1.88 (0.67-5.26) | .231 |
| Immunosuppression |  |  |  |
| - No | 32/67 (47.8) | NC | - |
| - Yes | 1/1 (100.0) |  |  |
| Charlson index |  |  |  |
| - <3 | 0/1 (0.0) | NC | - |
| - ≥3 | 33/67 (49.3) |  |  |
| 10-years expected survival^a^ |  |  |  |
| - ≥90% | 0/3 (0.0) | NC | - |
| - <90% | 33/65 (50.8) |  |  |
| **Clinical Presentation** |  |  |  |
| Clinical duration^b^ |  |  |  |
| - ≥7 days | 11/15 (73.3) | 1.00 (ref) | - |
| - <7 days | 16/31 (51.6) | 0.39 (0.10-1.49) | .167 |
| Fever |  |  |  |
| - No | 16/35 (45.7) | 1.00 (ref) | - |
| - Yes | 16/32 (50.0) | 1.19 (0.45-3.10) | .726 |
| Dry cough |  |  |  |
| - No | 16/38 (42.1) | 1.00 (ref) | - |
| - Yes | 16/29 (55.2) | 1.69 (0.64-4.49) | .290 |
| Wet cough |  |  |  |
| - No | 28/54 (51.9) | 1.00 (ref) | - |
| - Yes | 4/13 (30.8) | 0.41 (0.11-1.50) | .180 |
| Dyspnea |  |  |  |
| - No | 7/26 (26.9) | 1.00 (ref) | - |
| - Yes | 26/42 (61.9) | 4.41 (1.52-12.82) | **.006** |
| Diarrhoea |  |  |  |
| - No | 30/58 (51.7) | 1.00 (ref) | - |
| - Yes | 2/7 (28.6) | 0.37 (0.07-2.08) | .261 |
| Confusion |  |  |  |
| - No | 20/44 (54.4) | 1.00 (ref) | - |
| - Yes | 13/22 (59.1) | 1.73 (0.62-4.89) | .298 |
| Fatigue |  |  |  |
| - No | 27/52 (51.9) | 1.00 (ref) | - |
| - Yes | 5/12 (41.7) | 0.66 (0.19-2.36) | .523 |
| Myalgias-arthralgias |  |  |  |
| - No | 31/62 (50.0) | 1.00 (ref) | - |
| - Yes | 1/2(50.0) | 1.00 (0.06-16.71) | 1.000 |
| Anosmia-dysgeusia |  |  |  |
| - No | 31/60 (51.7) | 1.00 (ref) | - |
| - Yes | 1/3 (33.3) | 0.47 (0.04-5.44) | .544 |
| **Initial Assessment** |  |  |  |
| Oximetry at room air |  |  |  |
| - ≥94% | 10/22 (45.5) | 1.00 (ref) | - |
| - <94% | 22/40 (55.0) | 1.47 (0.52-4.17) | .473 |
| PaO2:FiO2 |  |  |  |
| - ≥300 | 22/45 (48.9) | 1.00 (ref) | - |
| - <300 | 11/21 (52.4) | 1.13 (0.36-3.59) | .833 |
| Respiratory rate |  |  |  |
| - ≤24 breaths/min | 6/20 (30.0) | 1.00 (ref) | - |
| - >24 breaths/min | 11/18 (61.1) | 5.13 (1.13-23.30) | **.034** |
| Systolic BP |  |  |  |
| - ≥100 mmHg | 29/60 (48.3) | 1.00 (ref) | - |
| - <100 mmHg | 2/6 (33.3) | 0.53 (0.09-3.14) | .488 |
| Diastolic BP |  |  |  |
| - ≥60 mmHg | 22/44 (50.0) | 1.00 (ref) | - |
| - <60 mmHg | 9/22 (40.9) | 0.69 (0.25-1.95) | .486 |
| Heart rate |  |  |  |
| - ≤100 beats/min | 23/51 (45.1) | 1.00 (ref) | - |
| - >100 beats/min | 9/16 (56.3) | 1.12 (0.35-3.64) | .850 |
| CURB65 |  |  |  |
| - <3 | 3/14 (21.4) | 1.00 (ref) | - |
| - ≥3 | 13/23 (56.5) | 4.77 (1.04-21.79) | **.044** |
| eGFR |  |  |  |
| - ≥60 mL/min/m^2^ | 7/23 (30.4) | 1.00 (ref) | - |
| - <60 mL/min/m^2^ | 27/45 (60.0) | 4.25 (1.41-12.84) | **.010** |
| Leukocytes |  |  |  |
| - ≤12940 per mm^3^ | 23/50 (46.0) | 1.00 (ref) | - |
| - >12940 per mm^3^ | 10/17 (58.8) | 1.68 (0.55-5.11) | .363 |
| Lymphocytes |  |  |  |
| - ≥610 per mm^3^ | 23/52 (44.2) | 1.00 (ref) | - |
| - <610 per mm^3^ | 10/16 (62.5) | 2.10 (0.67-6.64) | .206 |
| C-reactive protein |  |  |  |
| - ≤14.2 mg/dL | 20/50 (40.0) | 1.00 (ref) | - |
| - >14.2 mg/dL | 13/17 (76.5) | 4.88 (1.39-17.11) | **.013** |
| Procalcitonin |  |  |  |
| - ≤0.41 ng/mL | 17/44 (38.6) | 1.00 (ref) | - |
| - >0.41 ng/mL | 12/14 (85.7) | 9.53 (1.90-47.93) | **.006** |
| Ferritin |  |  |  |
| - ≤1300 mg/L | 13/43 (30.2) | 1.00 (ref) | - |
| - >1300 mg/L | 10/14 (71.4) | 5.77 (1.53-21.81) | **.010** |
| Lactate dehydrogenase |  |  |  |
| - ≤374.5 U/L | 15/43 (34.9) | 1.00 (ref) | - |
| - >374.5 U/L | 9/14 (64.3) | 3.36 (0.95-11.85) | .059 |
| D-dimers |  |  |  |
| - ≤2.7 | 17/47 (36.2) | 1.00 (ref) | - |
| - >2.7 | 12/15 (80.0) | 7.06 (1.74 - 28.57) | **.006** |
| Interleukin 6 |  |  |  |
| - ≤94 pg/mL | 4/25 (16.0) | 1.00 (ref) | - |
| - >94 pg/mL | 4/8 (50.0) | 5.25 (0.91-30.23) | .063 |
| Troponin T |  |  |  |
| - ≤55 ng/L | 17/42 (40.5) | 1.00 (ref) | - |
| - >55 ng/L | 10/15 (66.7) | 2.94 (0.85-10.14) | .088 |
| Brain natriuretic peptide |  |  |  |
| - ≤4794 pg/mL | 18/44 (40.9) | 1.00 (ref) | - |
| - >4794 pg/mL | 10/15 (66.7) | 2.89 (0.84-9.89) | .091 |
| Creatine phosphokinase |  |  |  |
| - ≤165 U/L | 20/47 (42.6) | 1.00 (ref) | - |
| - >165 U/L | 9/15 (60.0) | 2.03 (0.62-6.62) | .243 |
| Aspartate aminotransferase |  |  |  |
| - ≤54 U/L | 19/50 (38.0) | 1.00 (ref) | - |
| - >54 U/L | 13/17 (76.5) | 5.30 (1.51-18.65) | **.009** |
| Alanine aminotransferase |  |  |  |
| - ≤44 U/L | 22/49 (44.9) | 1.00 (ref) | - |
| - >44 U/L | 9/17 (52.9) | 1.38 (0.46-4.17) | .568 |
| Opacities of lung surface on X-rays |  |  |  |
| - ≤50% | 14/37 (37.8) | 1.00 (ref) | - |
| - >50% | 19/31 (61.3) | 3.29 (1.07-10.12) | **.038** |

Data shown as % unless specified otherwise. For units of the variable, please refer to Table 1. In bold, statistically significant differences. ^a^10-years expected survival derived from Charlson comorbidity index score. ^b^Days of symptoms before admission. OR: odds ratio, 95%CI: 95% confidence interval, NC: not calculable.
