# Supplementary material for: Fatality and risk features for prognosis in COVID-19 according to the care approach – a retrospective cohort study
Source: PLoS One. 2021 Mar 23;16(3):e0248869. doi: 10.1371/journal.pone.0248869 (PMC7987197; doi:10.1371/journal.pone.0248869)
Supplement: S2 Table — (DOCX) [file pone.0248869.s002.docx]

**S2 Table. Results of simple regression models for fatality, for whole population and for population with complete data in the final multiple model.**

|  | ***Global Population***  *N = 238* | | | ***Multivariate model population***  *N = 184* | | |  |
| --- | --- | --- | --- | --- | --- | --- | --- |
|  | *Death (n=13)* | *OR (95%CI)* | *p* | *Death*  *(n=8)* | *OR (95%CI)* | *p* | |
| Age |  |  |  |  |  |  | |
| - <65 | 3/149 (2.0) | 1.00 (ref) | - | 2/114 (1.8) | 1.00 (ref) |  | |
| - ≥65 | 10/89 (11.2) | 6.16 (1.65-23.04) | **.007** | 6/70(8.6) | 5.25 (1.03-26.78) | **.046** | |
| Charlson index |  |  |  |  |  |  | |
| - <3 | 3/142 (2.1) | 1.00 (ref) | **-** | 2/111 (1.8) | 1.00 (ref) |  | |
| - ≥3 | 10/96 (10.4) | 5.39 (1.44-20.13) | **.012** | 6/73 (8.2) | 4.88 (0.96-24.88) | .056 | |
| Confusion |  |  |  |  |  |  | |
| - No | 8/218 (3.7) | 1.00 (ref) | **-** | 6/178 (3.4) | 1.00 (ref) |  | |
| - Yes | 5/16 (31.3) | 11.93 (3.35-42.54) | **<0.001** | 2/6 (33.3) | 14.33 (2.18-94.16) | **.006** | |
| PaO2:FiO2 |  |  |  |  |  |  | |
| - ≥300 | 2/113 (1.8) | 1.00 (ref) | **-** | 3/143 (2.1) | 1.00 (ref) |  | |
| - <300 | 6/51 (11.8) | 7.40 (1.44-38.05) | **.017** | 5/41 (12.2) | 6.48 (1.48-28.40) | **.013** | |
| Systolic BP |  |  |  |  |  |  | |
| - ≥100 | 9/211 (4.3) | 1.00 (ref) | **-** | 5/172 (2.9) | 1.00 (ref) |  | |
| - <100 | 4/15 (26.7) | 8.08 (2.15-30.40) | **.002** | 3/12 (25) | 11.13 (2.29-54.09) | **.003** | |
| eGFR |  |  |  |  |  |  | |
| - ≥60 | 8/195 (4.1) | 1.00 (ref) | **-** | 4/152 (2.6) | 1.00 (ref) |  | |
| - <60 | 5/41 (12.2) | 3.25 (1.01-10.49) | **.049** | 4/32 (12.5) | 5.28 (1.25-22.39) | **.024** | |
| Lymphocytes |  |  |  |  |  |  | |
| - ≥790 | 5/172 (2.9) | 1.00 (ref) | **-** | 3/138 (2.2) | 1.00 (ref) |  | |
| - <790 | 8/64 (12.5) | 4.47 (1.21-16.51) | **.025** | 5/46 (10.9) | 5.48 (1.26-23.95) | **.024** | |
| Procalcitonin |  |  |  |  |  |  | |
| - ≤0.2 | 5/165 (3.0) | 1.00 (ref) | **-** | 4/144 (2.8) | 1.00 (ref) |  | |
| - >0.2 | 7/46 (15.2) | 5.74 (1.73-19.07) | **.004** | 4/40 (10) | 3.89 (0.93-16.31) | .063 | |
| Troponin T |  |  |  |  |  |  | |
| - ≤15 | 1/149 (0.7) | 1.00 (ref) | **-** | 1/132 (0.8%) | 1.00 (ref) |  | |
| - >15 | 7/58 (12.1) | 15.73 (1.79-138.56) | **.013** | 7/52 (13.5) | 20.38 (2.44-170.19) | **.005** | |
| Opacities of lung surface on X-rays |  |  |  |  |  |  | |
| - ≤50% | 7/169 (4.1) | 1.00 (ref) | **-** | 3/131 (2.3) | 1.00 (ref) |  | |
| - >50% | 6/69 (8.7) | 3.97 (1.26-12.53) | **.019** | 5/53 (9.4) | 4.44 (1.02-19.32) | **.047** | |

Data shown as n (%) unless specified otherwise. For units of the variable, please refer to Table 1. In bold, statistically significant differences. OR: odds ratio, 95%CI: 95% confidence interval.
